# In Situ Construction of ZIF-67-Derived Hybrid Tricobalt Tetraoxide@Carbon for Supercapacitor

**DOI:** 10.3390/nano12091571

**Published:** 2022-05-06

**Authors:** Hao Gong, Shiguang Bie, Jian Zhang, Xianbin Ke, Xiaoxing Wang, Jianquan Liang, Nian Wu, Qichang Zhang, Chuanxian Luo, Yanmin Jia

**Affiliations:** 1 NARI Corporation (State Grid Electric Power Research Institute), Nanjing 211106, China; 2395644259@foxmail.com (S.B.); 986461052@foxmail.com (X.K.); 1722495101@foxmail.com (N.W.); 894520714@foxmail.com (C.L.); 2 State Grid Electric Power Research Institute Wuhan NARI Corporation, Wuhan 430074, China; 3 State Grid Heilongjiang Electric Power Corporation, Academy of Science, Harbin 150030, China; 2754994719@foxmail.com; 4 School of Science, College of Science, Xi’an University of Posts and Telecommunications, Xi’an 710121, China; xxwang618@foxmail.com (X.W.); zqc029@foxmail.com (Q.Z.)

**Keywords:** ZIF-67, Co_3_O_4_ nanosheet, in situ growth, Co_3_O_4_@C, supercapacitor

## Abstract

The Co_3_O_4_ electrode is a very promising material owing to its ultrahigh capacitance. Nevertheless, the electrochemical performance of Co_3_O_4_-based supercapacitors is practically confined by the limited active sites and poor conductivity of Co_3_O_4_. Herein, we provide a facile synthetic strategy of tightly anchoring Co_3_O_4_ nanosheets to a carbon fiber conductive cloth (Co_3_O_4_@C) using the zeolitic imidazolate framework-67 (ZIF-67) sacrificial template via in situ impregnation and the pyrolysis method. Benefiting from the enhancement of conductivity and the increase in active sites, the binder-free porous Co_3_O_4_@C supercapacitor electrodes possess typical pseudocapacitance characteristics, with an acceptable specific capacitance of ~251 F/g at 1 A/g and long-term cycling stability (90% after cycling 5000 times at 3 A/g). Moreover, the asymmetric and flexible supercapacitor composed of Co_3_O_4_@C and activated carbon is further assembled, and it can drive the red LED for 6 min.

## 1. Introduction

Supercapacitors as next-generation energy storage devices have sparked intensive research interest in the fields of modern electronics and automobiles because of their safety, fast charging rate ultrahigh power density, etc. [1,2]. Due to Faradaic redox reactions in energy storage, pseudocapacitors generally show a higher capacitance than electric double-layer capacitors [3,4]. Among various pseudocapacitive electrode materials, transitional metal oxides with fast and reversible Faradic processes, which have been applied in pseudocapacitors [5,6,7,8], especially for Co_3_O_4_, have received extensive attention owing to the advantages of a high theoretical capacitance (of 3560 F/g), low cost, controllable morphology and favorable pseudocapacitive characteristics [3,9,10].

In spite of the various advantages of Co_3_O_4_, the intrinsic poor electronic conductivity and limited active sites may hinder their effective electrochemical reactions because of the agglomeration on supporting materials [11]. One-dimensional carbon materials are used in electrical and some electrochemical devices due to their superb electrical conductivity, effective electron transfer and chemical stability [12,13]. Therefore, the construction of the composite of Co_3_O_4_ with rich electro-active sites and carbonaceous materials is considered a prevalent strategy to ameliorate this issue [14,15]. Unfortunately, the surface functionalization of non-reactive carbon materials is usually necessary to activate the surface to obtain good sediment from these transition metal oxides, which results in an increase in interface resistance and a reduction in the active sites [16,17,18].

To alleviate this drawback, the metal–organic frameworks of ZIF-67 (zeolitic imidazolate framework-67), with high porosity and long-range ordering, proved to be promising precursors and self-sacrificial templates for Co_3_O_4_ growth via thermal decomposition [17,19]. The porous Co_3_O_4_, derived from ZIF-67 on carbon nanofibers matrix, exhibits excellent electrical conductivity due to abundant active sites [20,21]. In this regard, Yin et al. recently demonstrated that Co_3_O_4_@NiCo LDH nanosheets, derived from ZIF-67 by means of carbonization and electrodeposition, had an excellent specific capacitance (1708 F/g) [22]. Yue and co-workers fabricated a ZIF-67/Co_3_O_4_ nanoparticles@nitrogen-doped carbon nanofibers anode, which greatly enhanced the electrochemical performance of sodium-ion batteries [23]. Wang et al. selected Co_3_O_4_ particles/carbon aerogel derived from ZIF-67 as the electrode, and the specific capacitance value was up to 298.8 F/g [24]. Nonetheless, the supercapacitors electrode, composed of ZIF-67-derived porous Co_3_O_4_ nanosheets and the carbon-nanofibers-supporting matrix, is still in its infancy; more in-depth insights into such promising materials are urgently needed.

Herein, we propose a facile in situ impregnation method, combined with the pyrolysis process, at an optimized temperature for the synthesis of a ZIF-67-templated, highly oriented, porous Co_3_O_4_ nanosheet anchored on a carbon fiber cloth to form Co_3_O_4_@C as a supercapacitor electrode. The resultant hybrid structure possesses the complementary advantages of a conductive carbon substrate and highly ordered porous Co_3_O_4_ nanosheet, which can provide fast electrolyte diffusion and ion transport and many electroactive sites for an effective redox reaction. The synergistic effect of the porous Co_3_O_4_ nanosheet and a one-dimensional conductive carbon fiber endows the Co_3_O_4_@C electrode with a 251 F/g specific capacitance, and, after 5000 cycles, it still has a 90% retention rate and long-term cycle stability. Moreover, the practical application in lighting of the prepared electrode is further demonstrated with a flexible asymmetric supercapacitor made of Co_3_O_4_@C//active carbon, which can drive a red LED for 6 min.

## 2. Material Preparation and Test Methods

### 2.1. Materials

The chemicals, including Ni(NO_3_)_2_·6H_2_O (99%), Co(NO_3_)_2_·6H_2_O (99%), KOH (99%) 2-methylimidazole (99%) and N-methyl-2-pyrrolidone (99.5%), were supplied by Sinopharm Chemical Reagent Co., Ltd., Shanghai, China. Polyvinylidene fluoride (99.5%) and poly(acrylic acid) partial potassium salt (PAAK) (99%) were purchased from Shanghai Aladdin Biochemical Technology Co., Ltd., Shanghai, China. CC (WOS 1009) was purchased from CeTech Co., Ltd., Taiwan, China. Activated carbon (99.5%) and acetylene black (99.5%) were purchased from Fuzhou Yihuan Carbon Co., Ltd. (Fuzhou, China).

### 2.2. Fabrication of ZIF-67-Derived Co_3_O_4_@C Electrode

ZIF-67 was prepared following a reported method with minor modification [25]. Briefly, both Co(NO_3_)_2_·6H_2_O (0.58 g) and 2-methylimidazole (1.3 g) were individually dissolved in two beakers containing 40 mL deionized water. Next, the Co(NO_3_)_2_·6H_2_O solution was added into the solution of 2-methylimidazole for 0.5 h stirring. A piece of the hydrophilic pretreated carbon fiber cloth (1 × 2 cm^2^) with a purple color was vertically immersed in the mixture solution. The ZIF-67 crystal samples were in situ prepared and grown on the surface of a carbon cloth after being kept for 6 h at room temperature. After removing the resultant ZIF-67@C, it was treated by washing alternately and dried at 80 °C for 10 h. To define the most suitable calcination treatment on the Co_3_O_4_ formation, five different treatments at temperatures of 270 °C, 280 °C, 290 °C, 300 °C and 310 °C, with a 5 °C/min ramp rate for 1 h, were carried out in air. The resulting black film sample was denoted as Co_3_O_4_@C. The loading mass of Co_3_O_4_@C was about 1.3 mg/cm^2^, found by weighing the mass of the carbon cloth before growing ZIF-67 and after thermal treatment at 290 °C. The pure ZIF-67 and Co_3_O_4_ nanosheets were prepared without the introduction of carbon cloth.

### 2.3. Assembly of the Co_3_O_4_@C//AC Flexible Supercapacitor

The negative electrode was fabricated according to a common method. An electrode slurry was fabricated by stirring acetylene black, polyvinylidene fluoride and activated carbon (AC) at 1:8:1 mass ratio in the solvent of N-methyl-2-pyrrolidone. The carbon fiber cloth used as the current collector was immersed in the slurry for 2 min and dried for 10 h at a temperature of 80 °C. The gel electrolyte was fabricated by slowly adding 1 g PAAK to the 10 mL 1.78 M KOH aqueous solution under continuous stirring at room temperature until it became transparent. A piece of the filter paper (1 × 1 cm^2^) was employed to separate the negative and positive electrodes. The three Co_3_O_4_@C, filter paper and AC components were all immersed in the gel electrolyte, held for 30 s and successively assembled into the supercapacitor device.

### 2.4. Material Characterizations

The X-ray diffractometer showed the structure of the crystal (X-ray diffractometer) at 40 kV and λ = 0.1542 Å wavelength of Cu Kα radiation (D8 Advance, Bruker, MA, USA). Raman spectra were studied via the confocal laser micro-Raman spectrometer using a laser of He-Ne with a 532 nm excitation wavelength (RAMAN, Renishaw, London, UK). The information on morphology and elemental composition was obtained using a field-emission scanning electron microscope (FESEM, JSM7100F, JEOL, Tokyo, Japan) with the equipment of energy-dispersive X-ray spectroscopy (EDS). Furthermore, the valence state of the elements near the surface was distinguished by means of X-ray photoelectron spectroscopy (XPS, 250XI, Thermo ESCALAB, Waltham, MA, USA). The pore size distributions and the specific surface areas of the sample were also evaluated and measured with Brunauer–Emmett–Teller (BET) measurement equipment on the basis of the Barrett–Joyner–Halenda (BJH) method using 3H-2000BET-A apparatus (Beishide Co., Ltd., Beijing, China). Thermogravimetric (TG) analyses were conducted using a PerkinElmer Pyris Diamond TG/DTA instrument under air atmosphere. A DSC Q-100 (TA Instruments, New Castle, UK) was used to conduct differential scanning calorimetry (DSC) measurements.

### 2.5. Electrochemical Performance

These electrode samples were characterized and measured using a three-electrode system, where the Co_3_O_4_@C (1 × 1 cm^2^), Hg/HgO, 1 M KOH aqueous solution and platinum foil were used as the working electrode port, reference electrode port, electrolyte, and counter electrode site, respectively. Cyclic voltammetry (CV), galvanostatic charge/discharge (GCD) and electrochemical impedance spectroscopy (EIS) were analyzed in the Zahner CIMPS-2 electrochemical workstation. The LANHE battery testing system was used to study the cycling stability (CT2001A). The electrochemical tests of the assembled flexible Co_3_O_4_@C//AC supercapacitor, using the positive Co_3_O_4_@C electrode and the negative AC electrode, were investigated by similar analysis methods to those above. The mass loading of AC was calculated based on the following equation [22]:

$$\frac{m_{+}}{m_{-}}=\frac{C_{-}V_{-}}{C_{+}V_{+}}$$

where *m*_+_ and *m*_−_, *C*_+_ and *C*_−_, *V*_+_ and *V*_−_ are the mass loading, specific capacitance, potential window of positive electrode, and negative electrode, respectively. The mass loading of AC is about 2.2 mg cm^−2^. The specific capacitance was calculated according to the following equation [22]:

$$C=\frac{It}{mV}$$

where *I*, *t* and *V* are the discharge current, discharge time and potential window of the GCD measurement, respectively; *m* is the mass loading of active material.

## 3. Results and Discussion

**Scheme 1:**
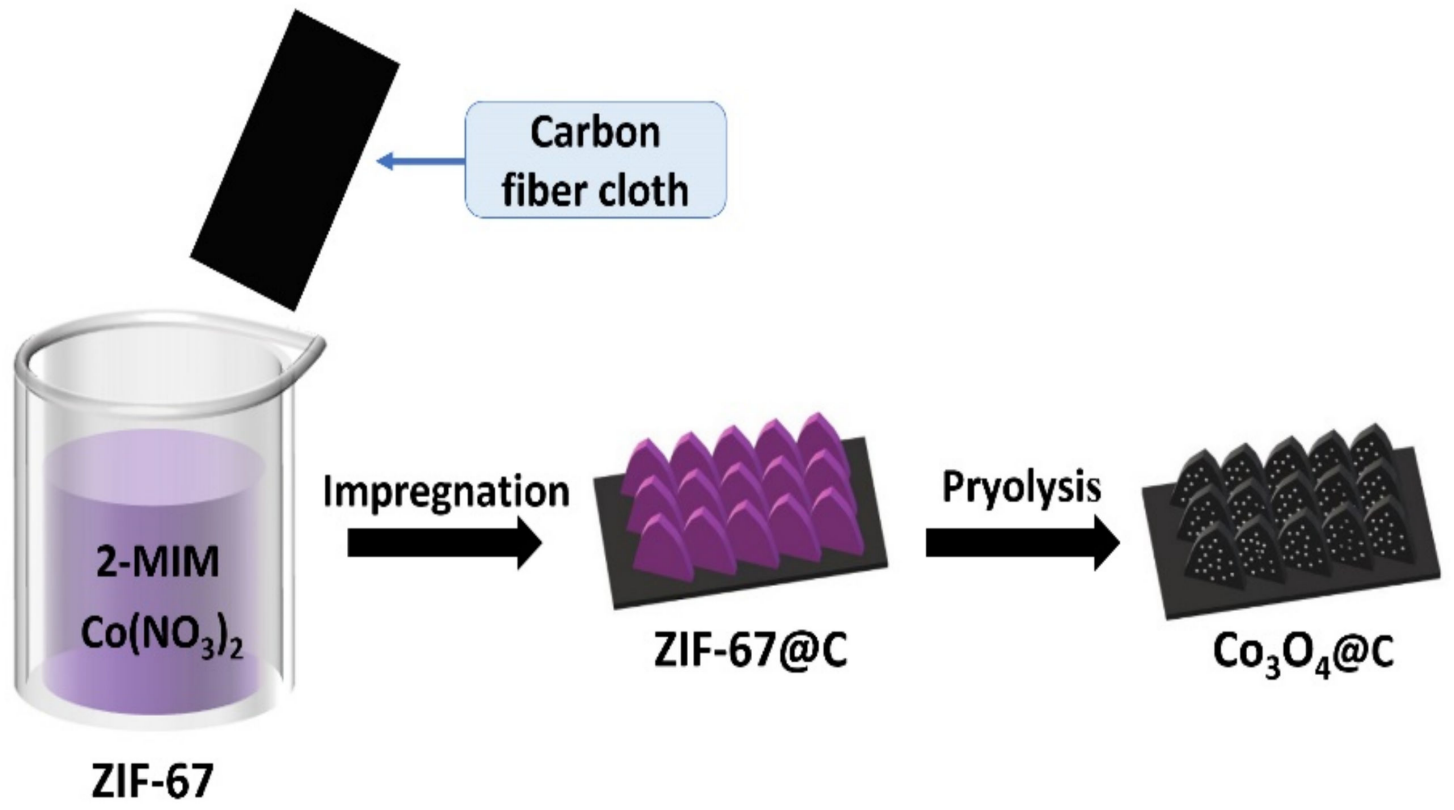
Synthesis process of ZIF-67-derived Co_3_O_4_@C electrode sample.

In Scheme 1, the fabrication procedure of the sample of ZIF-67-derived Co_3_O_4_@C electrode is schematically depicted. First, the ZIF-67 nanosheets were chemically compounded through a simple precipitation reaction by mixing a cobalt source of Co(NO_3_)_2_ and an organic linker of 2-methylimidazole (2-MIM) at room temperature. Second, the carbon fiber cloth was directly submersed in the above mixture solution to obtain the strong in situ ZIF-67 nanosheets grown on the carbon substrate, which changed from black to purple. Subsequent thermal treatment converted the ZIF-67 crystals, working as the Co precursor, to the final electrode of Co_3_O_4_@C. Different calcination temperatures of the ZIF-67@C in air were first investigated to obtain the optimal conversion temperature.

**Figure 1:**
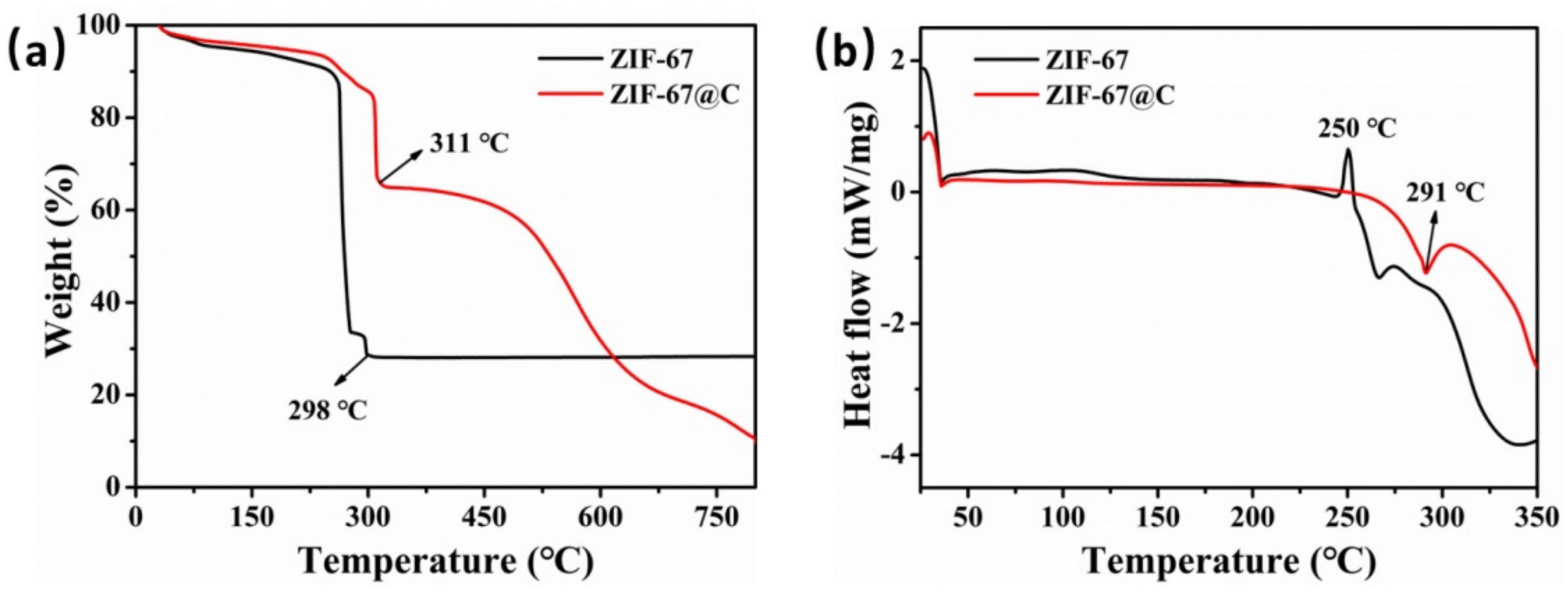
(**a**) TG curves and (**b**) DSC curves of the ZIF-67 sample powder and the self-assembled ZIF-67@C sample in air.

In order to determine the calcination temperature, TG-DSC analyses were conducted. As shown in Figure 1a, according to the result from the TG analysis, the weight loss of ZIF-67 powder is mainly divided into three parts. The first stage of weight loss (10%) occurred between room temperature and 250 °C, mainly due to the volatilization of absorbed water molecules and some residual molecular solvents. At the second stage of 250–276 °C and the third stage of 276–298 °C, 62% of weight loss may have originated from the products of H_2_O, NOx and CO_2_ gas in the thermal decomposition stage of the ZIF-67 precursors, which is supported by an exothermic peak of the DSC curve at 250 °C (Figure 1b) [26]. After 298 °C, the weight of ZIF-67 powder remained unchanged, indicating the thorough and complete conversion of ZIF-67 to Co_3_O_4_. Thus, the required calcination temperature should be above 250 °C. For the ZIF-67@C composite, the mass remained unchanged after 310 °C to some extent, indicating that the derived oxides obtained by pyrolysis were completely transformed and thermally stable. An endothermic peak in the DSC curve at 291 °C was generated by the thermal reduction of Co^3+^ to Co^2+^. The last loss took place after ~450 °C, originating from the combustion of the surrounding carbon matrix. Therefore, in order to obtain controllable Co^3+^/Co^2+^ contents and an adequate conversion of ZIF-67 to Co_3_O_4_, the calcination temperature should be in the range of 250–310 °C.

**Figure 2:**
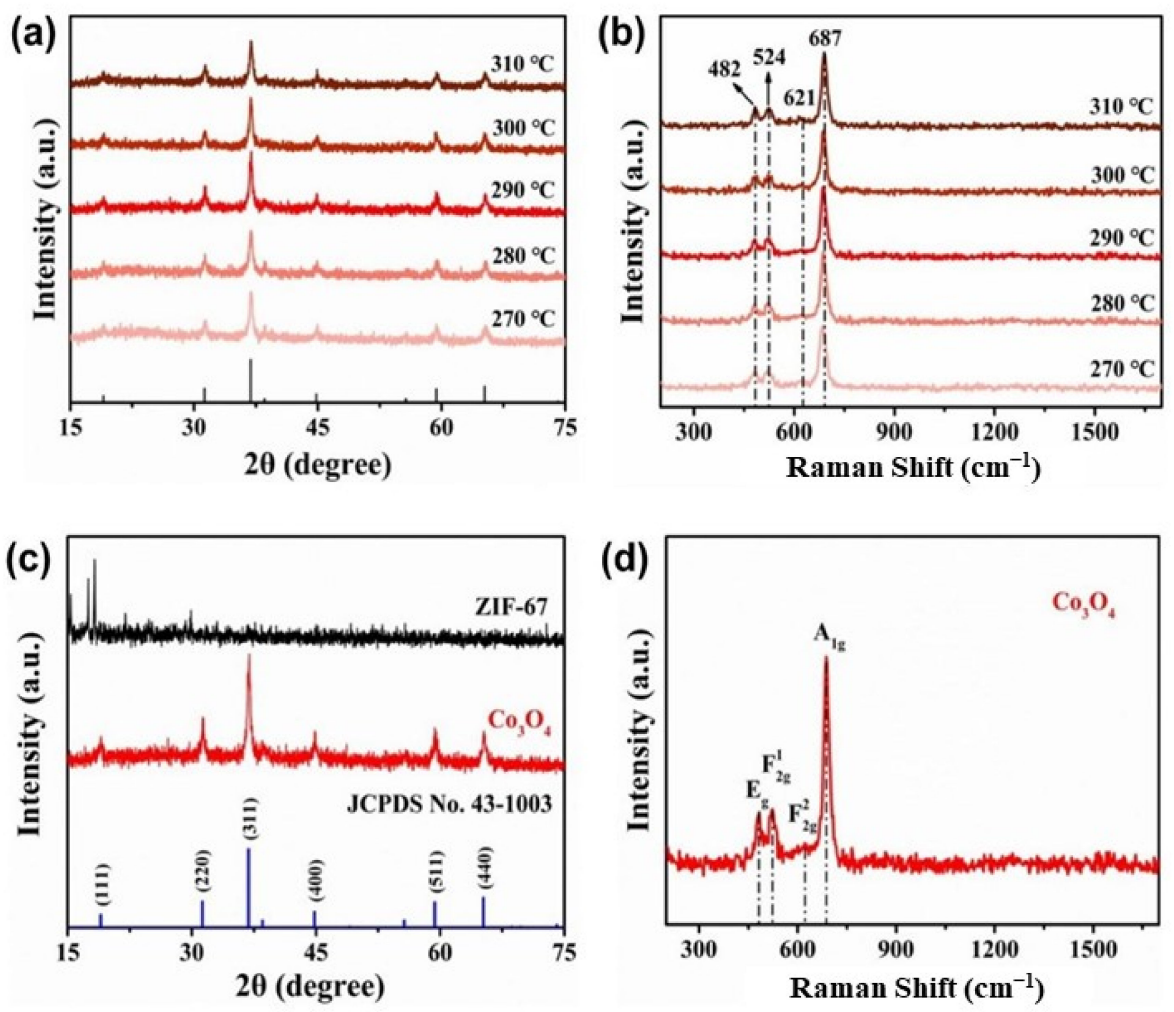
(**a**) The XRD pattern result of the ZIF-67-derived Co_3_O_4_ annealed at different temperatures, and (**b**) corresponding Raman spectra. (**c**) The XRD pattern result of the ZIF-67 and the ZIF-67-derived Co_3_O_4_. (**d**) The Raman spectra of the ZIF-67-derived Co_3_O_4_.

The structural transition from ZIF-67 to Co_3_O_4_ after thermal treatment at different temperatures, 270 °C, 280 °C, 290 °C, 300 °C and 310 °C, was characterized by XRD and Raman patterns (Figure 2a,b) [27,28,29,30]. Clearly, no secondary peaks in the XRD patterns and Raman patterns were observed in the five ZIF-67-derived Co_3_O_4_ samples, suggesting that the annealing temperature had a negligible influence on the phase structures. Figure 2c shows that the peaks of ZIF-67-derived Co_3_O_4_ at 19.01°, 31.27°, 36.84°, 44.81°, 59.35° and 69.74° correspond to the face-centered cubic Co_3_O_4_ phases of (111), (220), (311), (222), (400), (511) and (440) (JCPDS No. 43-1003), respectively. After pyrolysis treatment, these distinctive peaks of Co_3_O_4_ replaced the peaks of the ZIF-67 sample, showing that the ZIF-67 template was slowly transformed to Co_3_O_4_. As shown in Figure 2d, the crystal structure of Co_3_O_4_ was further investigated by Raman spectra. Four typical peaks of Co_3_O_4_, at around 482, 524, 621 and 687 cm^−1^, are consistent, respectively, with the Eg, F2g (2), F2g (1) and A1g vibration modes of the Co_3_O_4_ spinel crystals [31]. This confirms the conversion of ZIF-67 to Co_3_O_4_ since no carbon-related peak was detected.

**Figure 3:**
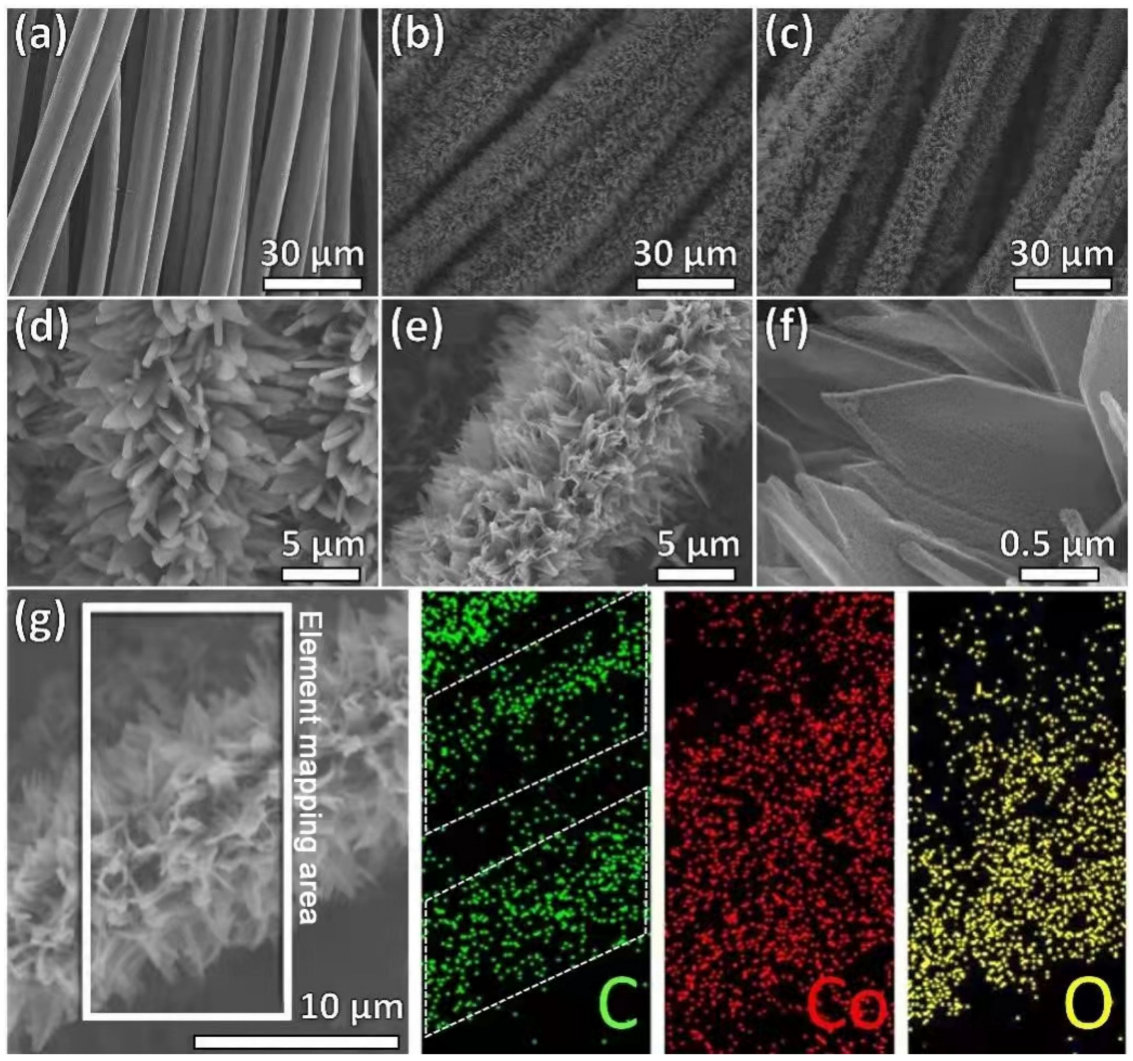
SEM pictures of (**a**) the carbon fiber cloth, (**b**) the pristine ZIF-67@C, (**c**) the ZIF-67-derived Co_3_O_4_@C, (**d**) the pristine ZIF-67@C at a high magnification, (**e**,**f**) ZIF-67-derived Co_3_O_4_@C at different magnifications (290 °C). (**g**) EDS elementary mapping results of C, Co and O in Co_3_O_4_@C.

Figure 3 depicts the morphology of the carbon fiber before and after ZIF attachment and ZIF-67-derived Co_3_O_4_@C. Through test results from SEM pictures [32,33,34,35], Figure 3a shows that the surface of carbon fiber was smooth and each fiber was separate from the other, each with a diameter of about 8 μm. Due to a good surface adhesion and uniform size, ZIF-67 grew vertically on each carbon fiber, with a good orientation and dispersion (Figure 3b,d). It is also evident that an individual ZIF-67 nanosheet with an average thickness of about 300 nm formed a leaf-like shape. The self-assembly of ZIF-67 on the flexible carbon fiber substrate avoids the use of adhesive, ensuring the nanosheet is fully in contact with the carbon fiber collector, which can not only reduce the load resistance of the electrode but also avoids a “dead surface” that cannot store energy. After the pyrolysis of ZIF-67, the nanosheet morphology was well-inherited but significantly shrank to ~224 nm due to the escape of COx and NOx [36], which is in agreement with the TG/DSC results (Figure 3c). High-resolution SEM images clearly show that the surface of Co_3_O_4_ became fairly rough and porous due to the release of gas during the calcination, indicating the increased specific surface area (Figure 3e,f). This can be verified by employing the BET measurement and BJH methods, where the specific surface area of Co_3_O_4_ is 66.32 m^2^/g, and the pore sizes are 9.25 nm higher than those of ZIF-67 (45.18 m^2^ g^−1^, 8.11 nm) (Table 1). The surface features of Co_3_O_4_ nanosheets are beneficial for accelerating the diffusion of electrolyte ions and for generating numerous electrochemical active sites for storage ions.

**Figure 4:**
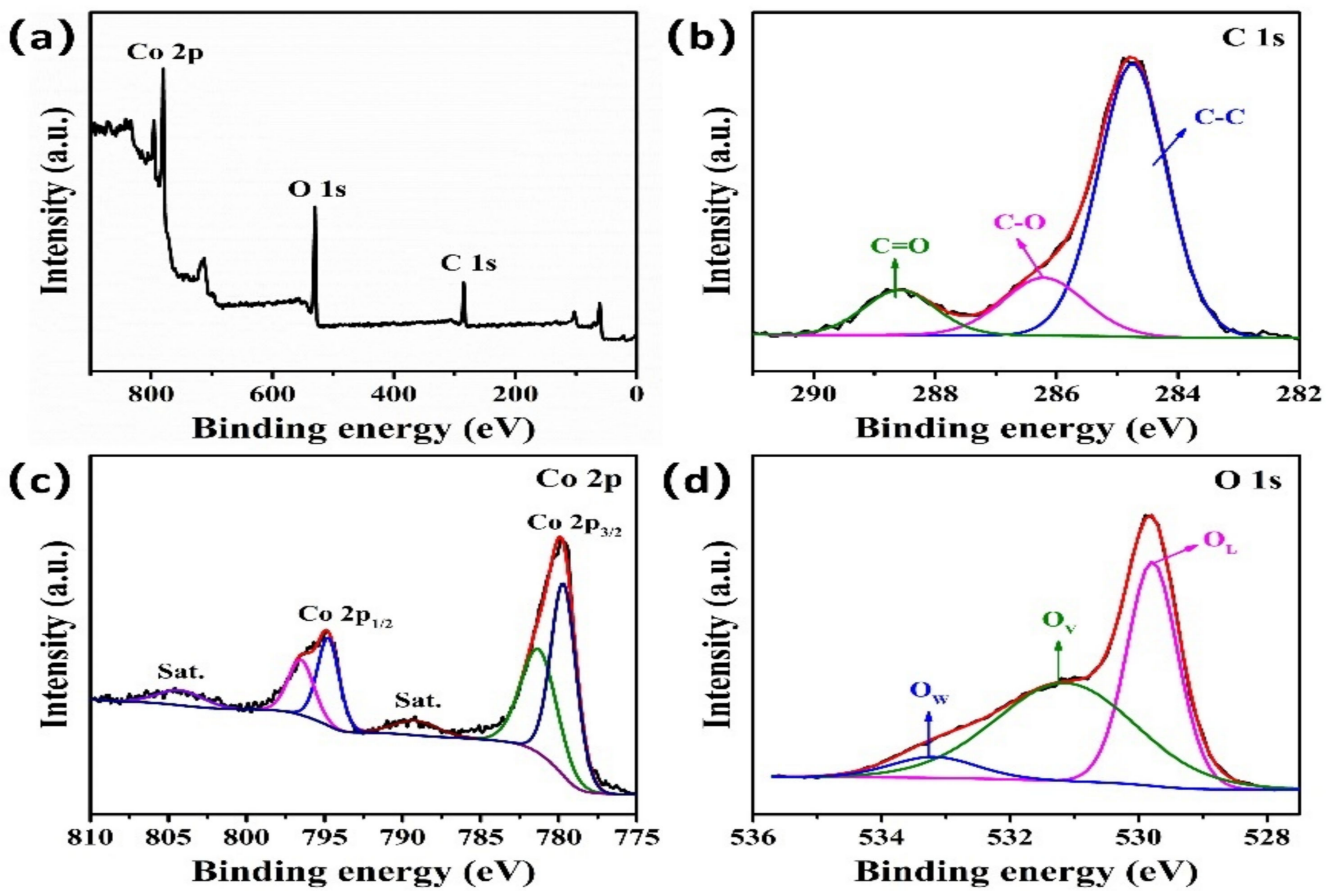
(**a**) The XPS survey spectra. The high-resolution XPS spectra of (**b**) the C 1s, (**c**) the Co 2p and (**d**) the O1s elements of ZIF-67-derived Co_3_O_4_.

The elemental distribution and chemical state of the self-assembly Co_3_O_4_ were further examined using EDS mapping and XPS analysis. As shown in the mapping images (Figure 3g), the C, Co and O elements were uniformly dispersed across the carbon fibers. Interestingly, the distribution of the C element was in perfect agreement with the shape of the carbon fibers; however, the Co and O elements were spread out over the entire fibers. These results confirm the attachment of ZIF-67-derived Co_3_O_4_ nanosheets to carbon fibers. Figure 4a presents the XPS survey spectra of ZIF-67-derived Co_3_O_4_ nanosheets. It can be seen that there is no N element belonging to the ZIF characteristic, while the partially retained C element suggests that the ZIF-67 frameworks were not destroyed. The remaining C element can improve the conductivity of Co_3_O_4_. As shown in Figure 4b, the C 1s XPS spectrum of 284.7 eV corresponds to the C-C bond, 286.2 eV corresponds to the C-O bond and 288.6 eV corresponds to the C=O bond [37,38]. In Figure 4c, the high-resolution spectrum shows a fitted and deconvoluted spectrum of Co 2p, suggesting that the CO^3+^ species correspond to the two peaks of 794.9 and 779.7 eV; the corresponding peaks of Co^2+^ are 796.6 and 781.3 eV. These two satellite peaks are considered to be Co^2+^ oxidation states [25]. The O 1s peak is deconvoluted into three parts: 533.2 eV belongs to lattice oxygen (OL, Co-O bond), 531.2 eV is ascribed to oxygen vacancy (OV), and 529.8 eV belongs to surface oxygen species (ow) (Figure 4d). The oxygen vacancies are reported to be generated accompanied with Co^2+^, which may be created by the difficult diffusion of atmospheric O_2_ into the lattice of Co_3_O_4_ in the presence of a carbon-reducing agent [11,39]. Thus, it is reasonable to conclude that part of the Co^3+^ ions, on the whole, were reduced to Co^2+^ and resulted in new oxygen vacancies, which were beneficial for accelerating ion transport [40]. These results clearly demonstrate the successful synthesis of Co_3_O_4_ nanosheets derived from the ZIF-67 sacrificial template.

**Figure 5:**
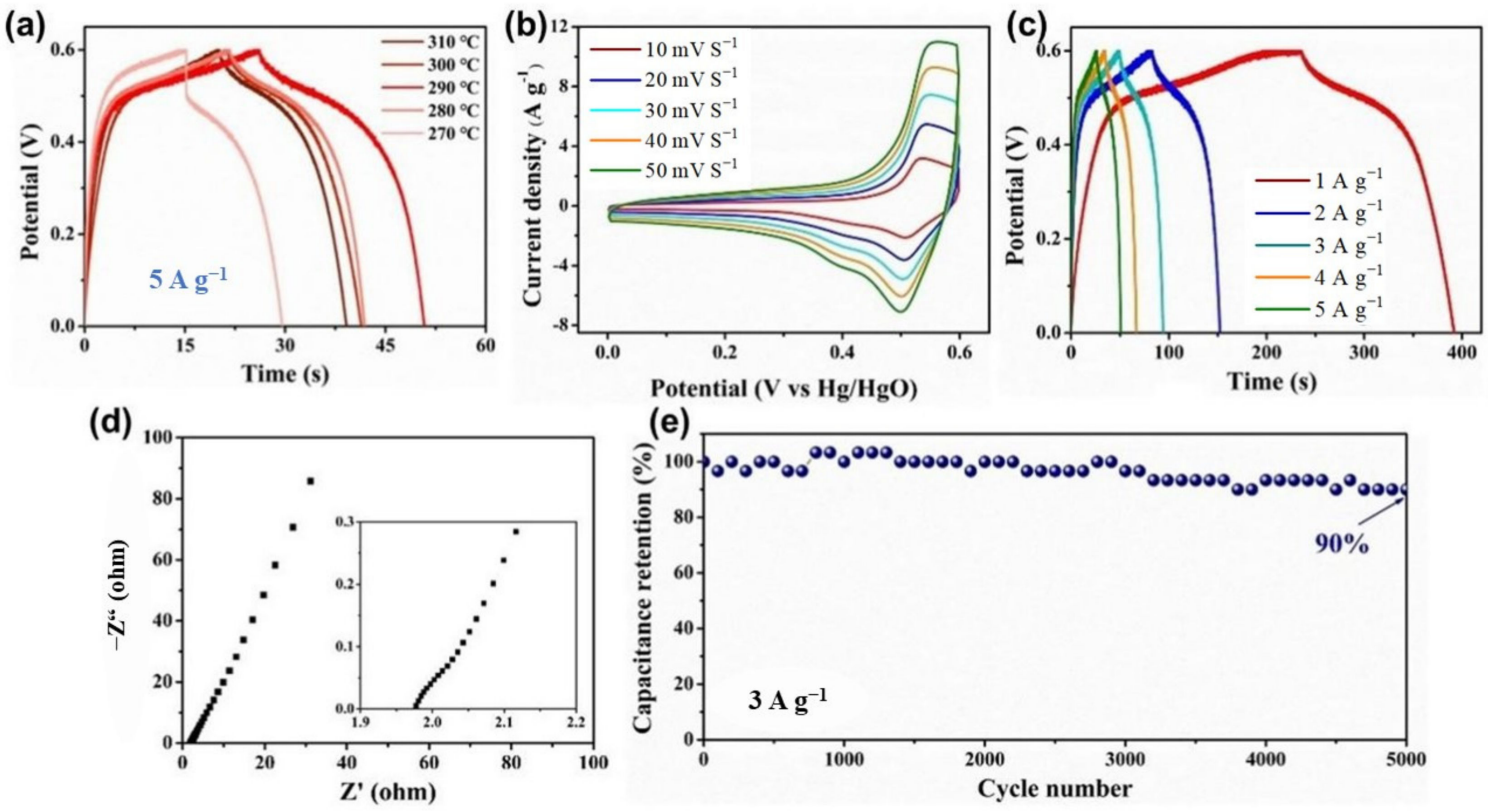
Electrochemical tests of the ZIF-67-derived Co_3_O_4_@C electrode. (**a**) Comparative GCD curves of ZIF-67-derived Co_3_O_4_@C electrodes with different annealing temperatures. (**b**) At a potential window of 0–0.6 V, CV curves for various scanning rates of Co_3_O_4_@C electrode (290 °C). (**c**) GCD curves under different current densities at the potential window of 0–0.6 V. (**d**) At 5 mV, amplitude Nyquist plots in a frequency range of 0.01 Hz–100 kHz (an enlarged part of the high-frequency region is in the insert). (**e**) At a constant current density of 3 A/g, the cycle function is tested.

Figure 5a shows the comparative GCD curves of Co_3_O_4_@C electrodes with different annealing temperatures. The result demonstrates that the Co_3_O_4_@C electrode annealed at 290 °C exhibited the maximum capacity. Thus, we focus on the Co_3_O_4_@C electrode sample synthesized at 290 °C in the following discussion. The scanning speed range was 10 to 50 mV/s.

Figure 5b presents the CV results of the Co_3_O_4_@C electrode (290 °C) at an operating potential of 0–0.6 V. Each curve associated with a reversible redox reaction of Co^3+^/Co^2+^ has obvious redox peaks in alkaline electrolyte, and these can be identified in the CV curve of the Co_3_O_4_@C electrode, which indicates the Faradaic pseudocapacitive behavior of the curves [14]. The increased integral area of CV curves with an increasing scan rate suggests the fast transport of ions and electrons. Furthermore, the well-retained shape indicates that the Co_3_O_4_@C electrode has a good rate performance. Figure 5c shows the GCD curves of the Co_3_O_4_@C electrode (290 °C), which shows an almost symmetrical shape at each charge–discharge curve, and this ensures high stability and superior reversibility of the electrochemical process. Moreover, when the current density is 1 A/g, the specific capacitance of the Co_3_O_4_@C electrode is 251 F/g, which remains 84% at 5 A/g (212 F/g). EIS was performed to analyze the conductivity, charge–transfer kinetics and ion diffusion behavior of Co_3_O_4_@C electrodes. The X-axis intercept and the arc diameter in the region of high frequencies are small, showing an equivalent series resistance (R_ESR_) of about 1.6 Ω. In the region of low frequencies, the slope of the incline line is large, which indicates the low charge transfer resistance and fast electrolyte diffusion (Figure 5d) [41]. This is because the interface formed between the Co_3_O_4_ nanosheet and the carbon fiber accelerates the transfer of charge; it speeds up the transfer and transport of ions and electrons. Furthermore, the Co_3_O_4_@C electrode can retain 90% of the highest capacitance after 5000 cycling times at 3 A/g, which shows that the electrode has ultrahigh stability (Figure 5e).

**Figure 6:**
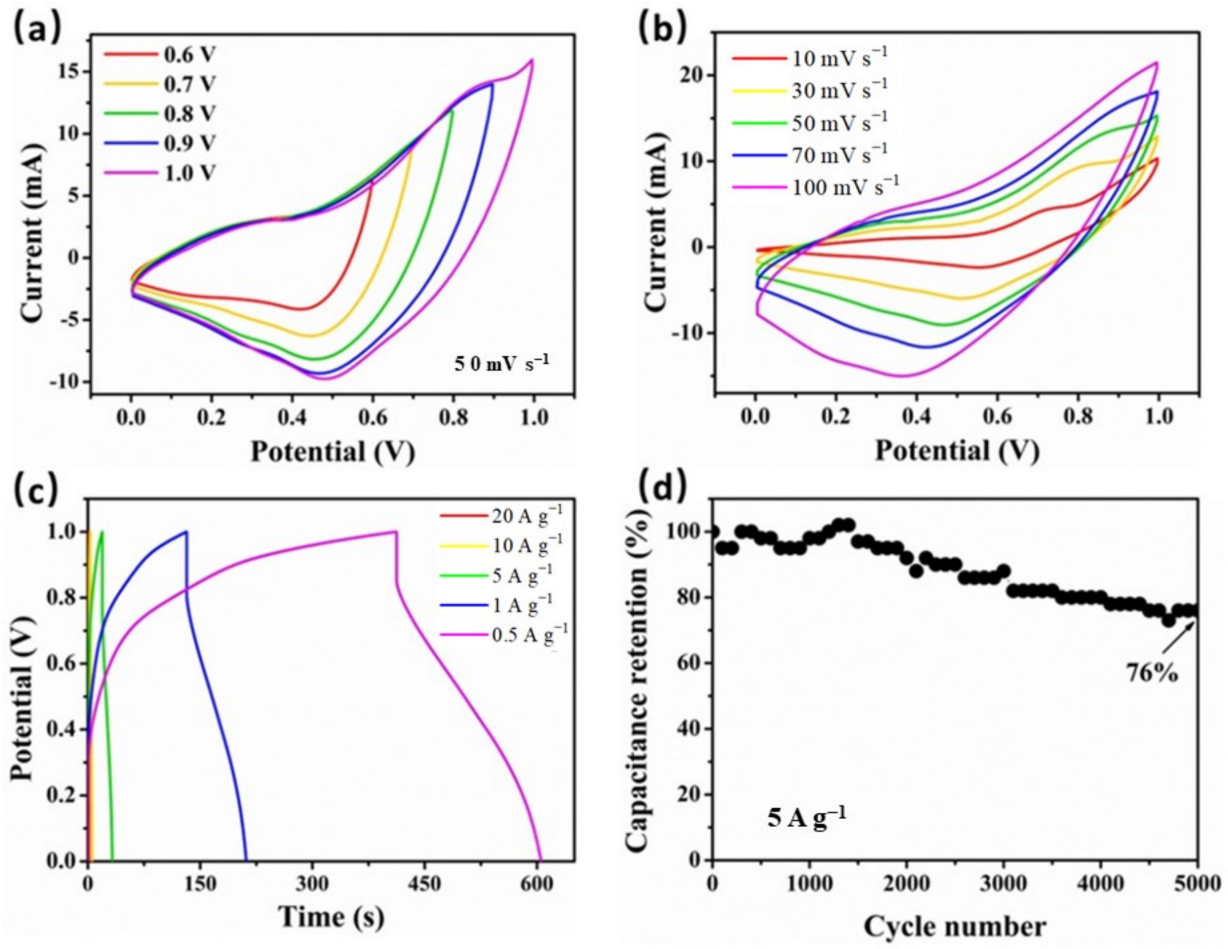
(**a**) The CV curves of Co_3_O_4_@C//AC asymmetric cell at various potential windows with 50 mV/s scan rate. (**b**) The CV curves for various scanning rates. (**c**) The GCD curves for different current densities. (**d**) The cycling capability.

An asymmetric solid-state supercapacitor (Co_3_O_4_@C//AC) was manufactured to further evaluate the practicality of the Co_3_O_4_@C electrode. A series of CV tests were performed at the same rate of 50 mV/s in the range from 0–0.6 V to 0–1 V to determine the appropriate potential window (Figure 6a). Curve polarization does not occur at each voltage window. Therefore, the operating potential window of the Co_3_O_4_@C//AC device is 1 V. With the increase in scanning rate, the shape of the CV curve has no obvious distortion and exhibits not only excellent stability but also fast charge/discharge features (Figure 6b). Its irregular shapes suggests a typical Faradaic redox process, which can be described as follows:

$$Co_{3}O_{4}+H_{2}O+OH^{-}↔3CoOOH+e^{-}$$

$$CoOOH+OH^{-}↔CoO_{2}+H_{2}O+e^{-}$$

Figure 6c shows the curves of GCD. The specific capacitance calculated from the GCD curves was 80 F/g at 1 A/g. The ~51% capacitance was retained at 5 A/g, and the capacitance retention of this device increased with the increase in cycling times. In Figure 6d, after 5000 cycles, it can be seen that 76% of capacitance is retained, displaying excellent stability.

The capacitance values and cycle times of relevant capacitor materials at different current densities (A/g) are summarized in Table 2. It can be seen from the data that the porous Co_3_O_4_ derived from ZIF-67 in the carbon nanofiber matrix has a good specific capacitance value and stability.

**Figure 7:**
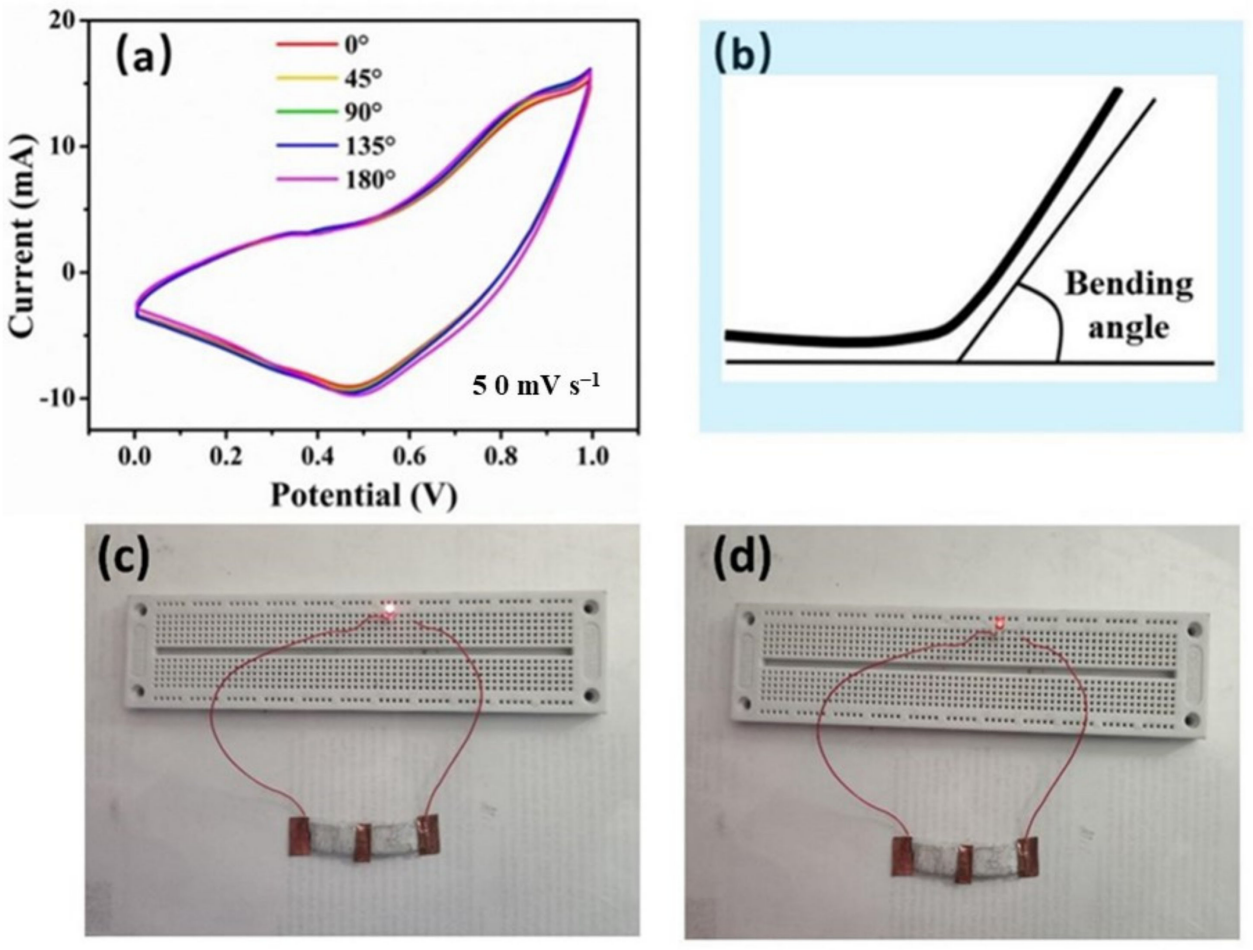
(**a**) The CV curves of the Co_3_O_4_@C//AC dissymmetric cell measured at varying bending angles from 0 to 180° and (**b**) the corresponding diagram. Pictures of driving red LED light with two series devices after (**c**) 1 min and (**d**) 6 min.

The flexibility of the assembled device was further tested (Figure 7a,b). The CV curves for these different bending angles from 0 to 180 degree at 50 mV s^−1^ remained almost the same, indicating that the device was highly flexible. This may have been caused by the synergy effect of the porous Co_3_O_4_@C electrode and the soft current collector. Moreover, the device did not leak any liquid materials nor cause a short circuit, confirming its excellent safety advantages. The practical application of the Co_3_O_4_@C//AC device was also explored by connecting two lighting devices in series. As illustrated in Figure 7c,d, the red LED (2 V, 0.06 W) was successfully lit for 6 min.

## 4. Conclusions

In this work, porous Co_3_O_4_ nanosheets were manufactured on carbon cloth via in situ impregnation and the pyrolysis method using the template of a ZIF-67 precursor. The Co_3_O_4_@C electrode displayed an acceptable specific capacitance of 251 F g^−1^ at 1 A/g and a superior rate capability with an 84% retention at 5 A/g, high cyclic stability, and 90% retention after 5000 cycles. These advantages are due to the highly ordered nanosheet structure of the material itself, and there is a synergistic interaction between Co_3_O_4_ and carbon cloth. These factors enhance the electrical conductivity and electroactive sites for Faradaic redox reactions. Our work reveals an effective approach and describes an in-depth investigation of the potential of using hybrid supercapacitor electrodes for energy storage applications.

## Figures and Tables

**Table 1:** Summary of the total specific surface areas (*S*_BET_), the sample’s pore diameters (*R*_pore_) and the sample’s pore volumes (*V*_pore_) of ZIF-67 and the ZIF-67-derived Co_3_O_4_.

Samples	*S*_BET_ (unit: m^2^/g)	*V*_pore_ (unit: cm^3^/g)	*R*_pore_ (unit: nm)
ZIF-67	45.18	0.01	8.11
Co_3_O_4_	66.32	0.11	9.25

**Table 2:** Comparison of specific capacitance and cycle times of relevant capacitor materials.

Materials	Rate	Specific Capacitance	Capacitance Retention/Cycle	Ref.
ZIF-67/Co_3_O_4_@C	1 A·g^−1^	251 F·g^−1^	90%/5000	This work
Co-MOF	0.6 A·g^−1^	206 F·g^−1^	97.5%/1000	[42]
Co_2_(OH)_2_C_8_H_4_O_4_/NF	2 mA·cm^−2^	13.6 F·cm^−2^	79.9%/1000	[43]
CoMn-MOF	5 mV·s^−1^	2.375 F·cm^−2^	85%/3000	[44]
CoFe-MOF	1 A·g^−1^	319.5 F·g^−1^	93.8%/3000	[45]
ZIF-67/Co_3_O_4_	5 A·g^−1^	190 F·g^−1^	72.45%/5000	[46]
Co_2_(H_2_O)(C_6_H_4_O_2_N)_4_/Co_3_O_4_	0.625 A·g^−1^	240 F·g^−1^	96.3%/2000	[47]
Co-MOF/NiCo_2_O_4_	2.5 mA·cm^−2^	1055 mF·cm^−2^	86.7%/20000	[48]
ZIF-67/C	20 mV·s^−1^	238 F·g^−1^	72%/200	[49]
ZIF-8@ZIF-67/NC@GC	2 A·g^−1^	270 F·g^−1^	100%/10000	[50]
ZIF-L(Zn)@ZIF-67/NC@GC@CNTs	2 A·g^−1^	252.1 F·g^−1^	91.2%/10000	[51]
ZIF-67/C@CNTs	10 mV·s^−1^	343 F·g^−1^	13%/1000	[52]

## Data Availability

Not applicable.
